# Mequindox-Induced Kidney Toxicity Is Associated With Oxidative Stress and Apoptosis in the Mouse

**DOI:** 10.3389/fphar.2018.00436

**Published:** 2018-05-01

**Authors:** Qianying Liu, Zhixin Lei, Jingchao Guo, Aimei Liu, Qirong Lu, Zainab Fatima, Haseeb Khaliq, Muhammad A. B. Shabbir, Muhammad Kashif Maan, Qinghua Wu, Menghong Dai, Xu Wang, Yuanhu Pan, Zonghui Yuan

**Affiliations:** ^1^National Reference Laboratory of Veterinary Drug Residues (HZAU) and MAO Key Laboratory for Detection of Veterinary Drug Residues, Huazhong Agricultural University, Wuhan, China; ^2^MOA Laboratory for Risk Assessment of Quality and Safety of Livestock and Poultry Products, Huazhong Agricultural University, Wuhan, China; ^3^College of Life Science, Yangtze University, Jingzhou, China; ^4^Department of Chemistry, Faculty of Science, University of Hradec Kralove, Hradec Kralove, Czechia; ^5^Hubei Collaborative Innovation Center for Animal Nutrition and Feed Safety, Wuhan, China

**Keywords:** mequindox, oxidative stress, Nrf2-Keap1, apoptosis, quinoxaline-di-*N*-oxides

## Abstract

Mequindox (MEQ), belonging to quinoxaline-di-*N*-oxides (QdNOs), is a synthetic antimicrobial agent widely used in China. Previous studies found that the kidney was one of the main toxic target organs of the QdNOs. However, the mechanisms underlying the kidney toxicity caused by QdNOs *in vivo* still remains unclear. The present study aimed to explore the molecular mechanism of kidney toxicity in mice after chronic exposure to MEQ. MEQ led to the oxidative stress, apoptosis, and mitochondrial damage in the kidney of mice. Meanwhile, MEQ upregulated Bax/Bcl-2 ratio, disrupted mitochondrial permeability transition pores, caused cytochrome c release, and a cascade activation of caspase, eventually induced apoptosis. The oxidative stress mediated by MEQ might led to mitochondria damage and apoptosis in a mitochondrial-dependent apoptotic pathway. Furthermore, upregulation of the Nrf2-Keap1 signaling pathway was also observed. Our findings revealed that the oxidative stress, mitochondrial dysfunction, and the Nrf2-Keap1 signaling pathway were associated with the kidney apoptosis induced by MEQ *in vivo*.

## Introduction

**FIGURE 1:**
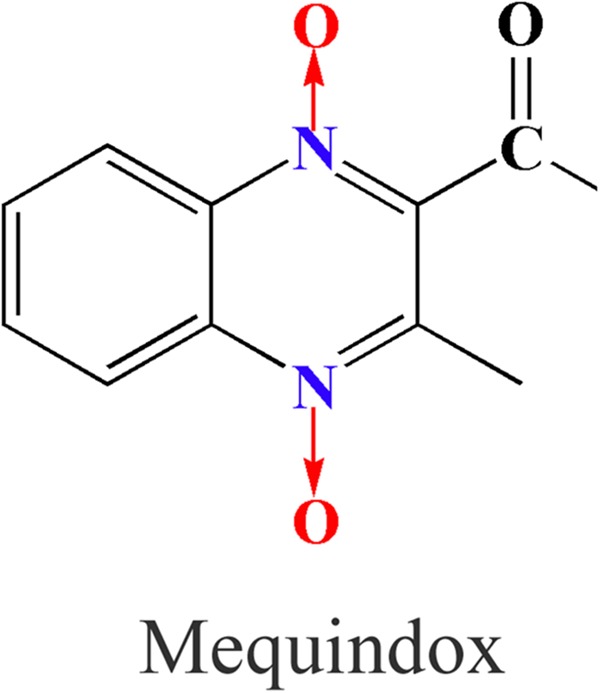
Chemical structure of mequindox (MEQ).

Quinoxaline-di-*N*-oxides (QdNOs), consisting of one or two acyclic chain moiety combined with quinoxaline ring, are best known as potential antibacterial agents (Wu et al., 2007; Vicente et al., 2009; Wang et al., 2011a, 2015a, 2016c; Cheng et al., 2015; Liu et al., 2016, 2017). Carbadox (CBX), olaquindox (OLA), quinocetone (QCT), and cyadox (CYA) were the classical members of QdNOs (Wang et al., 2015a,b; Zhang et al., 2015). Due to the potential genotoxic and carcinogenic effect, CBX and OLA had been banned in food-producing animals by European Commission since 1998 (Wang et al., 2016c). Mequindox (MEQ; 3-methyl-2-acetyl-*N*-1,4-dioxyquinoxaline,C_11_H_10_N_2_O_3_) (**Figure 1**), a relatively new QdNOs, can effectively improve growth and feed efficiency in animals for its better than other antimicrobial agents in treatment of swine dysentery (*Treponema hyodysenteriae*) (Liu et al., 2010; Ihsan et al., 2011, 2013a; Ding et al., 2012). As a synthetic antimicrobial agent, MEQ had significant antibacterial activities against both Gram-positive and Gram-negative species (Huang et al., 2015).

Previous studies demonstrated that some of the QdNOs such as OLA and CBX exhibited toxicity in the liver, kidney, and adrenal glands (Huang et al., 2010a,b; Ihsan et al., 2010, 2013a,b; Wang et al., 2016b; Liu et al., 2017). Early findings identified the adrenal glands as one of the main targets of QdNOs *in vitro* and *in vivo* (Huang et al., 2009, 2010a,b; Ihsan et al., 2010). MEQ was reported to reduce the output of adrenal aldosterone in pig adrenocortical cells (Huang et al., 2010b), and decreased serum aldosterone in rats (Huang et al., 2010a). The kidney has an important function in some unique physiology and metabolic pathways like the uptake, metabolism, and elimination of drugs (Lohr et al., 1998). Besides adrenal gland, kidney also plays an essential role in fluid-ion balance. However, the previous organ toxicity studies of MEQ only focus on the adrenal gland, while the kidney toxicity is commonly ignored. To date, only one study demonstrated the kidney damage on the male Wistar rats after exposure to MEQ (110 and 275 mg/kg) for up to 180 days, indicating that the kidney was another important target of MEQ (Huang et al., 2010a). However, the mechanism underlying kidney toxicity mediated by MEQ *in vivo*, especially regarding some linked signaling pathways was still not adequately understood.

A lot of evidence suggested that the oxidative stress was closely related to the damages caused by QdNOs, including apoptosis, DNA, and lipid damage (Chowdhury et al., 2004; Azqueta et al., 2007; Liu et al., 2012, 2017; Yang et al., 2013; Zhang et al., 2014; Wang et al., 2015a). The oxidative stress was also considered as the main factor in adrenal gland (Huang et al., 2009), liver (Wang et al., 2011b; Liu et al., 2017), spleen (Wang et al., 2011b), and testis toxicity (Ihsan et al., 2011) that caused by MEQ *in vivo*. The previous study concluded that MEQ-induced oxidative damage with the changes of reduced glutathione (GSH), superoxide dismutase (SOD), and malondialdehyde (MDA) in kidney (Huang et al., 2010a). Moreover, the oxidative stress was regarded as a molecular mechanism for MEQ to induce adrenal toxicity in H295R cells that originated from a human adrenocortical carcinoma (Wang et al., 2016b). Therefore, it was suspected that the oxidative stress may be involved in the toxicity in mice after chronic exposure to MEQ.

The QdNO-mediated oxidative stress is associated with a large number of biological responses and related cell signaling pathways. It was reported that the apoptosis invoked by OLA was always accompanied by the generation of ROS in renal tubular epithelial cells (HK-2 cells) (Li et al., 2015) and HepG2 cells that are from the liver (Zhang et al., 2011; Zhao et al., 2013, 2015). The apoptosis was observed in HepG2 cells after incubation with QCT (Zhang et al., 2013, 2015). However, whether the oxidative stress that triggered by MEQ-induced apoptosis in the kidney *in vivo* is unknown. Due to the wide use of MEQ in food animal production, it is of great significance to investigate the kidney toxicity mediated by MEQ *in vivo*.

In the present study, we investigated comprehensively the following parameters: the effects of MEQ exposure on body and kidney weight, morphological and ultrastructural changes in kidney; activity of the uric acid (UA), urea, creatinine (crea), blood urea nitrogen (BUN), GSH, total superoxide dismutase (T-SOD), MDA, 8-OHdG, and lactate dehydrogenase (LDH) in the serum; the mRNA expression of some genes in the kidney linked with apoptosis (e.g., caspase 3, caspase 8, caspase 9, and cychrome c), and correlated with oxidative stress (e.g., Nrf-2, GCLC, NQO1, HO-1, GSH-Px, and GST); the protein expression of cleaved caspase 3 and Nrf-2 in the kidney by immunohistochemistry, Western blot, and immunofluorescence assays. The study aimed at providing insight into molecular mechanism of MEQ-induced kidney toxicity *in vivo*, which will help to evaluate MEQ in its clinical use and improve the prudent use of QdNOs for public health.

## Materials and Methods

### Chemical Reagents

Mequindox (MEQ; C_11_H_10_N_2_O_3_, CAS No: 60875-16-3, purity 98%) was obtained from Beijing Zhongnongfa Pharmaceutical Co., Ltd. (Huanggang, China). SOD, GSH, 8-OHdG, MDA, and LDH kits were purchased from Nanjing Jiancheng Bioengineering Institute (Nanjing, China). Nrf-2 (D1Z9C) XP Rabbit mAb (12721T) and cleaved caspase 3 (Asp175) antibody (9661T) were purchased from Cell Signaling Technology (United States), and a reagent Western Blot Stripping Buffer (T7135A) was purchased from Takara (Japan). All other chemicals and reagents were of analytical grade and obtained from Sigma-Aldrich (St. Louis, MO, United States).

### Animals and Treatment

Recent studies revealed that the toxicity to liver (Liu et al., 2017) and testis (Liu et al., 2017a,b) were caused by MEQ in Kunming mice, while the kidney toxicity of MEQ in Kunming mice remains poor understood. Thus, the Kunming mice were chosen in this study. A total of 40 specific pathogen-free (SPF) Kunming mice (6–7 weeks old, weighing 30 ± 5g) were obtained from Center of Laboratory Animals of Hubei Province (Wuhan, China). The individual body weights of the mice were within ± 20% of the average. The mice were maintained in a room conditioned at 22 ± 3°C, a relative humidity of 50 ± 20%, and a 12-h light/dark cycle. The current study was approved by the Ethical Committee of the Faculty of Veterinary Medicine (Huazhong Agricultural University). Before treatment, the mice received basic feed and a standard diet from Nuvital Nutrients (Colombo/PR, Brazil), allowed to access to distilled water *ad libitum* for 7 days to evaluate for any signs of disease and weight gain.

According to the Organization for Economic Cooperation and Development (OECD) Guideline 453 and Procedures for toxicological assessment of food in China, the high-dose level should cause some toxic effect performance or damage, and the low-dose group may not show any toxic effects, but should be 1–3 times greater than the clinical dose (GB15193.17, 2003; OECD, 2009). In a previous sub-chronic and chronic toxicity study, MEQ in 110 mg/kg diet made an increase in plasma potassium (K^+^) level without growth inhibition, and this dose was determined to be no-observed-adverse-effect level (Ihsan, 2011). Therefore, the 110 mg/kg diet was selected as the high dose, and 55 mg/kg for the middle and 25 mg/kg for the low dose, respectively.

For the experiments, the mice were randomly divided into four groups (*n* = 10), including a control group treated with the basic diet without feed additives and three experimental groups treated with the same diet supplemented with 25, 55, and 110 mg/kg MEQ, respectively. The mice were housed five per group per sex in shoebox cages with hardwood shavings as bedding. Food and water were supplied *ad libitum* and the treatment period lasted for 11 months. During the experimental period, symptoms and mortality were observed and carefully recorded each day. In this study, the use of animals was in compliance with NIH Publication “The Development of Science Based Guidelines for Laboratory Animal Care” (NRC, 2004).

### Coefficients and Preparation of Kidney

Following an overnight fast, the mice were weighted and sacrificed. After weighting the body and kidneys, the coefficient of kidney to body weight was calculated as the ratio of kidney (wet weight, mg) to body weight (BW) (g). The kidneys were excised, rinsed in phosphate-buffered saline (PBS), and the left kidney from all mice (*n* = 10) was quickly frozen at -70°C.

### Histopathological Examination

All histopathological tests were performed using standard laboratory procedures. The right kidney from the mice (*n* = 5) was preserved in 10% neutral-buffered formalin. After fixation, the kidneys were embedded in paraffin blocks, then sliced into 5 μm in thickness and placed onto glass slides with hematoxylin–eosin (HE) staining. Slides were observed and the photos were taken using an optical microscope (Olympus BX 41, Japan) for morphological alterations.

### Observation of Nephrocyte Ultrastructure by Transmission Electron Microscope

The right kidney from other mice (*n* = 5) was fixed by 2.5% glutaraldehyde in 0.1 mol/dm^3^ cacodylate buffer for 4 h, then followed by wash three times with 0.1 mol dm cacodylate buffer (pH 7.2–7.4) and put in 1% osmium tetraoxide for 1 h. A graded series of ethanol (75, 85, 95, and 100%) was used to dehydrate the specimens and embedded in Epon 812. Ultra-thin sections (70 nm) were contrasted with lead citrate for 10 min and uranyl acetate for 30 min, and then observed with an H-7650 TEM (Hitachi, Japan). The nephrocyte apoptosis was judged according to the nuclear morphology changes (e.g., chromatin fragmentation and condensation).

### TUNEL Assay

The right kidney from the mice (*n* = 5) was performed on 4-μm paraffin sections using antigen retrieval for 10 min of boiling in 10 mM citrate buffer (pH 6.0). They were fixed in 4% paraformaldehyde (pH 7.4) at -20°C for 3 min. After washing with PBS, the sections were permeabilized with 0.1% Triton X-100. Then, the samples were washed in PBS and incubated with a terminal deoxynucleotide transferase-mediated dUTP nick end labeling (TUNEL) reagent containing terminal deoxynucleotidyl transferase and fluorescent isothiocyanate dUTP. After incubation, they were stained with 1 μg/ml DAPI for 30 min to investigate the cell nucleus by UV light microscopic observations (blue). The kidney samples were analyzed in a drop of PBS under a fluorescence and UV light microscope. All morphometric measurements were observed by at least three independent individuals in a blinded manner.

### Biochemical Analysis

For biochemical analysis, the serum aliquots were obtained by placed the blood samples in serum tubes at temperature of 24°C for approximately 30 min. After clotting, the blood tubes were centrifuged at 3000 rpm for 10 min using a Himac CR 21 G centrifuge (Hitachi, Tokyo, Japan). Supernatants were separated out and then stored at -20°C for further analysis. Kidney functions were determined by serum activities of UA, urea, crea, and BUN. All biochemical assays were performed using a Synchron CX4 Clinical System (Beckman Coulter, Brea, CA, United States) according to the manufacturer’s protocol (Beijing Leadman Biochemistry Technology Co., Ltd., Beijing, China).

### Oxidative Stress Assay

The effects of MEQ on the activity of 8-OHdG, MDA, GSH, T-SOD, and LDH in the serum were examined. Assays of 8-OHdG, MDA, T-SOD, and GSH levels were performed using commercial kits. The release of LDH was assessed using Synchron Clinical System CX4 (Beckman Coulter, Brea, CA, United States) according to the manufacturer’s directions (Beijing Leadman Biochemistry Technology Co., Ltd., Beijing, China). The data were analyzed according to the manufacturer’s instructions.

### RNA Extraction and qPCR

Total RNA from the left kidney from all mice (*n* = 10) was extracted using the Trizol Reagent according to the manufacturer’s instructions. RNA (1 μg) was reverse transcribed to cDNA with the use of ReverTra Ace^TM^ First Strand cDNA Synthesis Kit (Promega, United States). Synthesized cDNA was used for quantitative real-time polymerase chain reaction (Bio-Rad, United States) by SYBR^®^ Premix Ex Taq^TM^ RT-PCR kit (Takara, CodeDRR041 A, Japan). The mRNA expression of the apoptotic cytokines (e.g., caspase 3, caspase 8, caspase 9, cytochrome c, Bcl-2, and Bax), and oxidative stress-related genes (e.g., Nrf-2, HO-1, GCLC, NQO-1, GST-Px, and GST) were determined by real-time quantitative reverse transcriptase-polymerase chain reaction (RT-PCR). Mice specific primers were designed using Primer Express Software according to the software guidelines (**Table 1**). The primers were manufactured by Nanjing Genescript Co., Ltd. (Nanjing, China). For the 25 μL PCR reaction, 12.5 μL SYBR^®^ Premix Ex Taq^TM^, 1.0 μL of each primer (10 μm), 2.0 μL of cDNA, and 8.5 μL Rnase Freed H_2_O were mixed together.

**Table 1:** PCR primers used in the gene expression analysis.

Gene name	Description	Primer sequence (5′–3′)	Primer size (bp)
β-actin	mβ-actin-F	CTGTCCCTGTATGCCTCTG	221
	mβ-actin-R	TTGATGTCACGCACGATT	
Nrf-2	mNrf-2-F	TCCTATGCGTGAATCCCAAT	103
	mNrf-2-R	GCGGCTTGAATGTTTGTCTT	
NQO1	mNQO1-F	TTCTGTGGCTTCCAGGTCTT	104
	mNQO1-R	TCCAGACGTTTCTTCCATCC	
GCLC	mGCLC-F	ATGTGGACACCCGATGCAGTATT	200
	mGCLC-R	GTCTTGCTTGTAGTCAGGATGGTTT	
HO-1	mHO-1-F	GACAGAAGAGGCTAAGACCGC	213
	mHO-1-R	TGGAGGAGCGGTGTCTGG	
GST	mGST-F	CCGCTCTTTGGGGCTTTAT	191
	mGST-R	GGTTCTGGGACAGCAGGGT	
GSH-Px	mGSH-Px-F	GAAGTGCGAAGTGAATGG	224
	mGSH-Px-R	TGTCGATGGTACGAAAGC	
Caspase-8	mCaspase-8-F	ATCTGCTGTATCCTATCCCACG	180
	mCaspase-8-R	AGGCACTCCTTTCTGGAAGTTAC	
Caspase-9	mCaspase-9-F	GCGGTGGTGAGCAGAAAGA	190
	mCaspase-9-R	CCTGGGAAGGTGGAGTAGGA	
Caspase-3	mCaspase-3-F	CTGACTGGAAAGCCGAAACTC	203
	mCaspase-3-R	GACTGGATGAACCACGACCC	
Cytochrome c	mCytochrome c-F	CATCCCTTGACATCGTGCTT	250
	mCytochrome c-R	GGGTAGTCTGAGTAGCGTCGTG	
Bcl-2	mBcl-2-F	TGTGGTCCATCTGACCCTCC	224
	mBcl-2-R	ACATCTCCCTGTTGACGCTCT	
Bax	mBax-F	GGATGCGTCCACCAAGAAG	194
	mBax-R	CAAAGTAGAAGAGGGCAACCAC	

For GSH-Px, HO-1, GCLC, and Caspase 8, the cycling conditions were as follows: step 1, 30 s at 95°C; step 2, 45 cycles at 95°C for 5 s, 55°C for 30 s; step 3, dissociation stage. For Nrf-2, NQO1, GST, caspase 3, Bcl-2, Bax, and cytochrome c, the cycling conditions were as follows: step 1, 30 s at 95°C; step 2, 45 cycles at 95°C for 5 s, 60°C for 30 s; step 3, dissociation stage. For caspase 9, the cycling conditions were as follows: step 1, 30 s at 95°C; step 2, 45 cycles at 95°C for 5 s, 62°C for 30 s; step 3, dissociation stage. In this study, the housekeeping gene β-actin was used as internal calibrator reference gene for expression profiling of apoptotic and oxidative stress-related genes.

Following amplification, the authenticity of the amplified product by its specific melting temperature (Tm) was verified by a melting curve analysis with the complementary computer software. The threshold cycle of gene of interest and housekeeping gene (HK), and the difference between their *C*t values (Δ*C*t) were counted. Relative quantitative analyses of gene expression were calculated using 2^-ΔΔCt^ data analysis method in accordance with the previous literatures (Huang et al., 2009; Wang et al., 2016b; Liu et al., 2017).

### Western Blotting

The Nrf-2 (D1Z9C) XP^®^ Rabbit mAb (12721T) and cleaved caspase 3 (Asp175) antibody (9661T) were purchased from Cell Signaling Technology. Total protein of the tissues was extracted according to the manufacturer’s recommended protocol (Vazyme, Nanjing, China). Phenylmethylsulfonyl fluoride (PMSF) was added into radio immunoprecipitation assay (RIPA) lysis buffer, and the final concentration of PMSF was 1 mM. Total protein from the kidney was isolated in a preparation of RIPA lysis buffer, and then a sonic oscillator was used to break the cells or organelles. Next, it was centrifuged at 12000 g for 30 min at 4°C. The protein concentrations were determined using the BCA Protein Assay Kit (Shanghai Beyotime Biotechnology Co., Ltd., Shanghai, China). Samples with equal amounts of protein (50 μg) were loaded for 1-dimensional SDS–PAGE using 12% separating gel and 5% stacking gel. The separated proteins were electrophoretically transferred to a polyvinylidene fluoride (PVDF) (Minipore) membranes in Trans-Blot Cells (Liuyi, Beijing, China). The membranes were blocked with 5% nonfat milk in Tris-buffered saline containing 0.1% Tween-20 (TBS-T) for 1 h and were then immunoblotted with the primary antibody (anti-cleaved caspase 3 antibody at a dilution of 1:1000; anti-Nrf-2 antibody at a dilution of 1:1000) overnight at 4°C. The membranes were washed for 10 min 3 times with TBS-T and then were incubated with horseradish peroxidase-conjugated secondary anti-IgG antibody (diluted 1:5000) (Beyotime Inst. Biotech, Peking, China) at room temperature for 1 h. The membranes were washed for 10 min three times with TBS-T. Immunoreactive bands were visualized with a chemiluminescent substrate (ECL-Plus, Minipore) for 2–5 min. Images were captured with a LAS-4000 luminescent image analyzer (Fujifilm, Tokyo, Japan).

### Immunohistochemical Assay

The kidney was dissected out and fixed in 10% neutral-buffered paraformaldehyde, and paraffin blocks were embedded on appropriate glass slides for processing. These were placed into the oven at 58°C, for 10 min, followed by deparaffinization in xylol, rehydration in alcohol at decreasing concentrations, and washing in distilled water and PBS (0.1 M sodium phosphate buffer, pH 7.2) for 5 min. The endogenous peroxidase was blocked with a 3% hydrogen peroxide solution for 30 min, and then washed in distilled water and PBS for 15 min (3 times, 5 min each). The slides were placed in citrate buffer and subjected to steam heat recovery, maintained at 95–100°C for 30 min, and they were then naturally cooled. The glass slides were then washed with PBS (3 times, 5 min each) and incubated with 5% bovine serum albumin (BSA) for 1 h, in a moist chamber at 37°C. After that, the BSA was discarded, and the glass slides were incubated with appropriate primary antibodies (Nrf-2, 1:500; cleaved caspase 3, 1:300), diluted according to the manufacturer’s instructions (Santa Cruz or Millipore, United States), overnight at 4°C, in a moist chamber. Negative controls were processed without using the primary antibodies. The glass slides were then washed with PBS (3 times, 5 min each) and incubated with the biotinylated secondary antibody for 1 h at 37°C in the moist chamber. After another wash in PBS (5 times, 5 min each), they were incubated in 0.1% domain antibody (DAB) solution (in 3% hydrogen peroxide). The glass slides were counter stained with hematoxylin for 2 min, and then washed in running water for 10 min. Finally, the glass slides were washed in distilled water, dehydrated in alcohol (at increasing concentrations), diaphanized in xylol, and mounted on Entelan^®^ for optic microscopy examination.

### Immunofluorescence Assay

The kidney samples were performed on 4 μm paraffin sections of kidney using antigen retrieval for 10 min of boiling in 10 mM citrate buffer (pH 6.0). They were fixed in 4% paraformaldehyde (pH 7.4) at -20°C for 3 min. After washing 4 times in PBS, the sections were permeabilized with 0.1% Triton X-100, exposed to the blocking solution (PBS/3% BSA) and incubated with the primary antibodies cleaved caspase 3 and Nrf-2 at 4°C overnight. After four washes in PBS, the sections were incubated with secondary fluorescently labeled antibodies Dylight 594 antibodies for 45 min and then were washed 3 times in PBS. Nuclei were stained using DAPI. Fluorescent images were taken using an AX70 widefield microscope (Olympus). All morphometric measurements were observed by at least three independent individuals in a blinded manner.

### Statistical Analysis

All results are expressed as means ± SD. Statistical analysis was examined by SPSS 15.0 software. Group difference was assessed by one-way analysis of variance (ANOVA) followed by least significance difference (LSD) test. The *p* < 0.05 was considered statistically significant.

## Results

### MEQ-Induced Kidney Injury in Mouse

#### Body Weight and Kidney Coefficients

**FIGURE 2:**
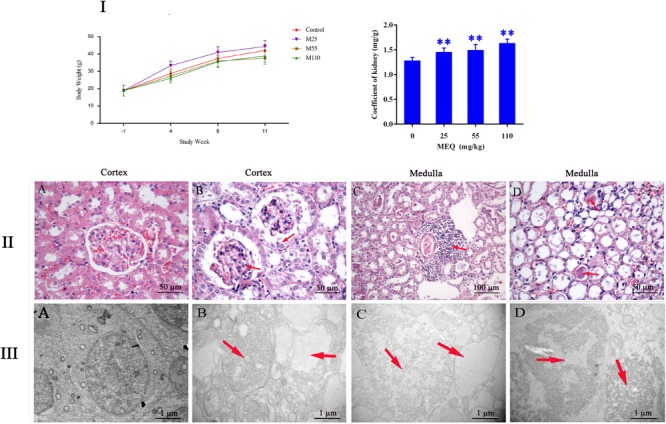
**(I)** Effects of MEQ on body weight (g) and kidney coefficients (mg/g) in different groups. **(II)** Selected microphotographs of kidney (200×, 400×). **(A)** Kidney from the control group (400×). **(B)** Kidney from the 25 mg/kg MEQ group showing swelling and hyperplasia of the renal capsule wall of cell (400×). **(C)** Kidney from the 55 mg/kg MEQ group exhibited aggregation of the lymphocyte into a group around the central veins (200×). **(D)** Kidney from the 110 mg/kg MEQ group, renal tubular epithelial cells was marked with degeneration and necrosis (400×). **(III)** Ultrastructure of kidney in mice after the administration of MEQ for 11 months (Scale bar = 1 μm). **(A)** The kidney from control group showing normal nucleus and completed mitochondrial membrane; **(B)** the kidney from 25 mg/kg MEQ group showing vacuolization of organelles and nuclei; **(C)** the kidney from 55 mg/kg MEQ group showing cell swelling and vacuolization; **(D)** the kidney from 110 mg/kg MEQ group showing nuclei dissolution and cells loss. ^∗^*p* < 0.05, and ^∗∗^*p* < 0.01. Values represent means ± SD (*n* = 10).

During administration, all the mice were growing and the clinical behaviors, such as food and water consumption in the MEQ-treated groups were as normal as in the control group. The coefficient of kidney to body weight was expressed as milligrams (wet weight of kidneys, mg)/(grams body weight, g). The final body weight and kidney coefficient of mice after administration of MEQ for 11 months were shown in **Figure 2I**. Compared with the controls, significant reductions in body weight were observed in 25, 55, and 110 mg/kg groups (*p* < 0.05 or *p* < 0.01), while the significant increase in kidney coefficients were noted in 25, 55, and 110 mg/kg MEQ groups (*p* < 0.01).

#### Histopathological Evaluation

The frame of reference as to where in the kidney these slides were obtained in the **Supplementary Figure S1**. As shown in **Figure 2II**, the significant histopathological changes in kidneys were observed in MEQ-treated groups. The glomerular capillaries exhibited expansion, and the renal capsule wall of cell showed swelling and hyperplasia in 25 mg/kg MEQ group (**Figure 2IIB**). Lymphocyte aggregated into a group around the central veins in 55 mg/kg MEQ group (**Figure 2IIC**). In 110 mg/kg MEQ group, renal changes were characterized by degeneration and necrosis of renal tubular epithelial cells with the appearance of protein casts in the tubular lumen (**Figure 2IID**). This result suggested that the kidney damage was induced by MEQ *in vivo.*

#### Ultrastructural Changes and Apoptosis Induced by MEQ

**FIGURE 3:**
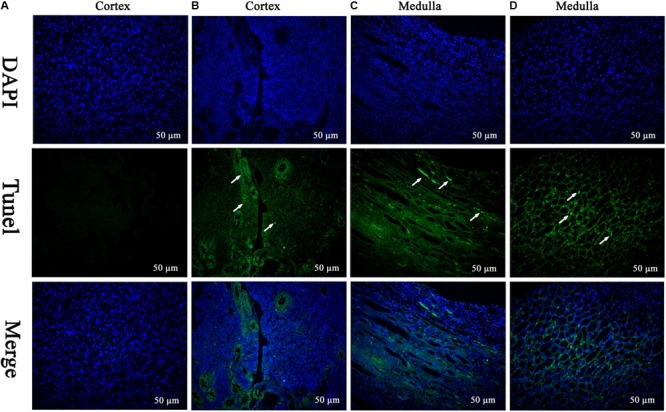
TUNEL staining of kidney tissue. Cell nuclei (Blue), TUNEL-positive cells (Green) (Scale bar = 50 μm). The green spots represent TUNEL-positive cells and the white arrows indicate the apoptotic region. **(A)** The kidney from control group showing normal cells; **(B–D)** the kidney from MEQ-treated groups showing tissue lesion and apoptosis.

The TEM analysis and TUNEL staining were carried out to investigate the ultrastructural changes and apoptosis of kidney induced by MEQ (**Figures 2III, 3**). The kidney in the control group presented normal nuclear morphology and completed mitochondrial membrane (**Figure 2IIIA**). In MEQ-treated groups, cells loss, cell swelling, nuclei dissolution, and mitochondria appeared vacuolization were noted (**Figures 2IIIB,C,D**). Additionally, the obvious apoptosis was also observed in MEQ-treated groups (**Figures 3B,C,D**). These findings revealed the obvious apoptosis and mitochondria damage caused by MEQ in the mouse.

### The Changed Serum Biochemical Levels and Oxidative Stress Induced by MEQ

**FIGURE 4:**
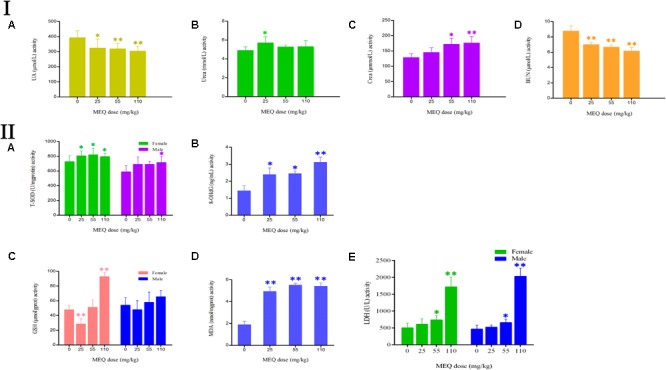
**(I)** Effects of MEQ on the activity of **(A)** UA, **(B)** Urea, **(C)** Crea, and **(D)** BUN in the serum of mice after administration of MEQ for 11 months. **(II)** Effects of MEQ on the levels of **(A)** T-SOD, **(B)** 8-OHdG, **(C)** GSH, **(D)** MDA, and **(E)** LDH in serum of mice after administration of MEQ for 11 months. ^∗^*p* < 0.05, and ^∗∗^*p* < 0.01. Values represent means ± SD (*n* = 10).

The effect of MEQ on the serum biochemical parameters was presented in **Figure 4I**. In comparison with the control group, the UA and BUN had shown a greatly decreased in all the MEQ-treated groups (*p* < 0.05 or *p* < 0.01). The significant increased levels of urea and crea was found in 25 mg/kg MEQ group (*p* < 0.05), and in 55 and 110 mg/kg MEQ groups (*p* < 0.05 or *p* < 0.01), respectively. The changed serum biochemical parameters demonstrated the chronic kidney disease occurred in mice after exposure to MEQ.

As shown in **Figure 4II**, the levels of 8-OHdG and MDA were markedly increased in the MEQ-treated groups (*p* < 0.05 or *p* < 0.01). The levels of T-SOD were significantly increased on females after exposure to MEQ at 25, 55, and 110 mg/kg groups (*p* < 0.05), and on males at 110 mg/kg (*p* < 0.05), respectively. For the effects of MEQ on the activity of GSH, the significant increased levels were observed in the female at 110 mg/kg group (*p* < 0.01), and significant decreased in the male at 25 mg/kg group (*p* < 0.01). These results show that the oxidative stress was triggered in mice after chronic administration of MEQ.

### MEQ-Induced Apoptosis and Activation Nrf2-Keap1 Signaling Pathway

**FIGURE 5:**
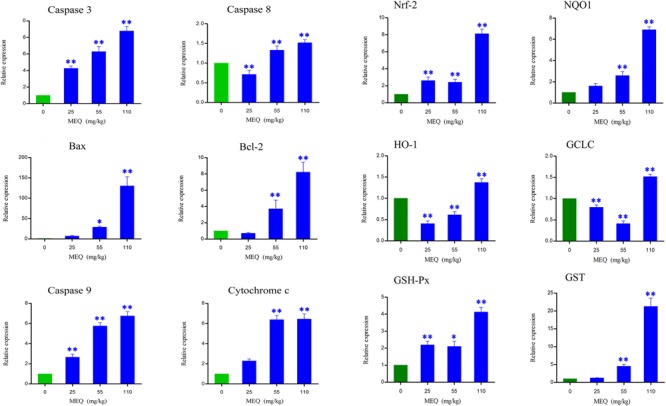
Alterations of Bax, Bcl-2, Caspase 3, Caspase 8, Caspase 9, Cychrome c, Nrf-2, NQO1, HO-1, GCLC, GSH-Px, and GST mRNA expression by RT-PCR in mouse kidney after administration of MEQ for 11 months. ^∗^*p* < 0.05, and ^∗∗^*p* < 0.01. Values represent means ± SD (*n* = 10).

Apoptosis plays an important role in both the physiological process of kidney growth and in different human renal diseases. To determine the role of apoptosis signal pathway in the mouse kidney after administration of MEQ for 11 months, real-time quantitative RT-PCR was used to demonstrate the changes of the genes including caspase 3, caspase 8, caspase 9, Bcl-2, Bax, and cytochrome c (**Figure 5**). With increased MEQ doses, there was a significant increase in caspase 3 and caspase 9 (*p* < 0.01). Exposure to MEQ significantly induced the expression of cytochrome c, Bcl-2, and Bax in 55 and 110 mg/kg groups (*p* < 0.05 or *p* < 0.01). A significant increased expression of caspase 8 was observed in 55 and 110 mg/kg MEQ groups (*p* < 0.01), and a marked reduction expression of caspase 8 was observed in 25 mg/kg MEQ group (*p* < 0.01).

**FIGURE 6:**
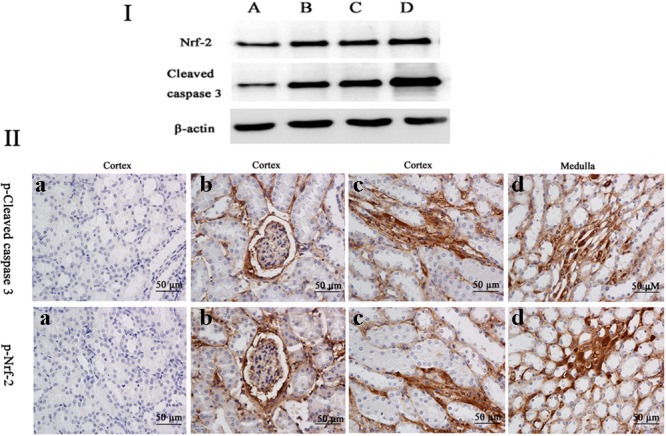
**(I)** The protein levels of phosphorylated Nrf-2 and cleaved caspases 3 were determined by Western blotting. β-actin was used as an internal control. **(II)** The protein expression of cleaved caspase 3 and Nrf-2 were detected by immunohistochemical assays (the positive reaction of anti-cleaved caspase 3 and anti-Nrf-2 antibody were brown); **(a)** the kidney from the control group, **(b–d)** the kidney from M25, M55, and M110 groups, respectively, showed a positive reaction in the glomerulus and medulla kidney.

**FIGURE 7:**
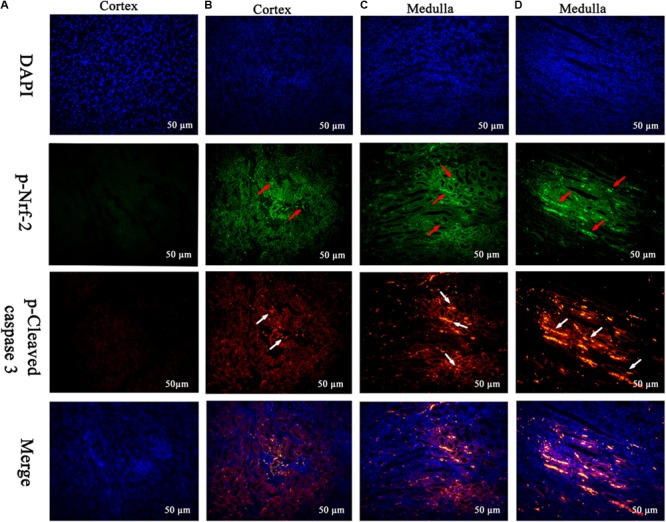
Translocation of the Nrf-2 from the cytoplasm into the nucleus and the expression of cleaved caspase 3 were evaluated by immunofluorescence (Scale bar = 50 μm). Blue spots represent cell nuclei, green spots represent Nrf-2 staining, and red spots represent cleaved caspase 3 staining. The integrated option density (IOD) of DAPI was used as an internal control. **(A)** Indicates the kidney from the control group, and **(B–D)** indicate the kidney from M25, M55, and M110 groups, respectively.

The frame of reference as to where in the kidney these slides were obtained in the **Supplementary Figures S2–S7**. Cleaved caspase 3 is a well-characterized cell apoptotic marker, the protein level of cleaved caspase 3 was detected by Western blot (**Figure 6I**), immunohistochemical analyses (**Figure 6II**), and immunofluorescence assay (**Figure 7**), indicating that compared to the control group, treating with MEQ caused increased cleaved caspase 3 expression. Taken together, these results suggested that the apoptosis induced by MEQ in kidney, and the mitochondrial dysfunction and caspase activation was involved in apoptosis mediated by MEQ.

Some oxidative stress-related genes (e.g., Nrf-2, HO-1, NQO1, GCLC, GST-Px, and GST) were determined using real-time quantitative RT-PCR (**Figure 5**). Exposure to MEQ significantly induced the expression of Nrf-2 and GSH-Px in the MEQ-treated groups (*p* < 0.05 or *p* < 0.01). An increased expression mRNA level of NQO1 and GST were observed on the doses of 55 and 110 mg/kg MEQ group (*p* < 0.01). There was a significant increased expression of HO-1 and GCLC in 110 mg/kg MEQ group (*p* < 0.01), and an obvious reduction expression of HO-1 and GCLC in 25 and 55 mg/kg MEQ groups (*p* < 0.01).

Accordingly, Western blot (**Figure 6I**), immunohistochemical analyses (**Figure 6II**), and immunofluorescence assay (**Figure 7**) also showed that MEQ-treated groups significantly increased the expression of Nrf-2 compared with those in the control group. These results showed that the activation of Nrf2/Keap1 signaling pathways mediated by MEQ might be a cellular protection response to oxidative stress in kidney.

## Discussion

The role of oxidative stress associated with organ toxicity mediated by QdNOs was extensively studied in rats (Huang et al., 2009, 2010a; Ihsan et al., 2010, 2011; Wang et al., 2011b; Yu et al., 2013, 2014) and mice (Wang D. et al., 2011; Zhao et al., 2011; Liu et al., 2017). The kidney has been identified as the main toxic organ target of QdNOs including MEQ. However, unlike other toxicities caused by QdNOs, their effect on kidney is not well studied. The results of present study demonstrated that the oxidative damage was triggered by MEQ, which might be responsible for apoptosis and mitochondrial dysfunction in kidney of mice after the administration of MEQ for 11 months. Additionally, MEQ-induced apoptosis via upregulated Bax/Bcl-2 ratio, caused cytochrome c release and a cascade activation of caspase. Furthermore, the Nrf2-Keap1 signaling pathway might be a protective response to the apoptosis in mice kidney caused by MEQ. This study is of great significance to evaluate MEQ in its clinical use and improve the prudent use of QdNOs for public health.

The kidney was reported to be an important target organ of MEQ, and the main characters of the kidney damage included the cell loss, cell swelling, nuclei shrinkage, hemorrhages, cell atrophy, and vacuolation in the Wistar rats after chronic exposure to MEQ (Huang et al., 2010a). In the present study, a significant reduction in body weight and a significant increase in the kidney coefficients were observed in mice after treatment of MEQ at doses of 25, 55, and 110 mg/kg for 11 months. In serum biochemical analysis, MEQ increased the levels of urea and crea, and reduced the concentration of BUN and UA. The altered levels of these parameters indicated chronic kidney disease in mice. Histopathological evaluation showed obvious kidney damage in MEQ-treated groups, including expansion of glomerular capillaries, swelling, degeneration, and necrosis of renal tubular epithelial cells. In the TUNEL staining and TEM analysis, the obvious apoptosis and ultrastructural changes including cells loss, cell swelling, nuclei dissolution, and vacuolization of mitochondria were caused by MEQ. The histopathological and TEM observations revealed that MEQ caused damage to cortex and medulla of the kidney. The effect of MEQ was primary on renal tubule and collecting duct. These results demonstrated that apoptosis in kidney occurred after chronic oral administration of MEQ, which confirmed the earlier findings that the kidney was an important toxicity organ target for MEQ *in vivo*.

Previous studies documented that the oxidative stress was closely related to the *in vitro* toxicity caused by QdNOs, such as cytotoxicity, adrenal toxicity, genotoxicity, and apoptosis (Liu et al., 2016, 2017). Long-term MEQ treatment–induced endocrine and reproductive toxicity via oxidative stress with the significant changed levels of SOD, GSH, MDA, and 8-OHdG in male Wistar rats (Ihsan et al., 2011; Liu et al., 2016). A recent study revealed that the oxidative stress played a critical role in the liver toxicity after chronic exposure to MEQ for 11 months (Liu et al., 2017). Herein, our results showed that the levels of MDA, 8-OHdG, T-SOD, GSH, and LDH were significantly increased in the MEQ-treated groups (**Figure 5**), suggesting an imbalance of redox in mice. This finding revealed that chronic exposure to MEQ invoked oxidative stress in mice, and the oxidative stress was closely related to the organ toxicity mediated by MEQ *in vivo*.

Cleaved caspase 3 is a well-characterized cell apoptotic marker and caspase 9 is a biomarker of mitochondrial apoptosis pathway. The multimeric complex formation of cytochrome c, caspase 8, and caspase 9 activates downstream caspases, leading to apoptosis cell death (Alabsi et al., 2016). Simultaneously, the Bcl-2 family proteins function as central regulators of apoptosis in mammals (Alabsi et al., 2016). In this study, MEQ significantly increased the protein expression of cleaved caspase 3 and mRNA expression of caspase 3, suggesting that the apoptosis was induced by chronic exposure to MEQ in the kidney of mice. Additionally, the mRNA expression of caspase 8 and caspase 9 were significantly increased, indicating a mitochondrial apoptosis pathway invoked by chronic exposure to MEQ. Furthermore, the expression of cytochrome c was increased in MEQ-treated groups, demonstrating that cytochrome c was possibly released into the cytosol, and subsequently, induced the changed of mitochondrial permeability transition pores. In response to a variety of apoptosis stimuli, over-expression of Bcl-2 or Bax blocks cytochrome c release (Li and Wang, 2006). In our study, Bcl-2 and Bax gene expression were overwhelmingly increased and the ratio of Bax/Bcl-2 markedly elevated by MEQ. These results were consistent with the ultrastructural changes of kidney under TEM observation. Taking together, these findings indicated that the mitochondrial-dependent apoptotic pathway was triggered by MEQ, and MEQ upregulated Bax/Bcl-2 ratio, caused cytochrome c release and a cascade activation of caspase, eventually induced apoptosis.

**FIGURE 8:**
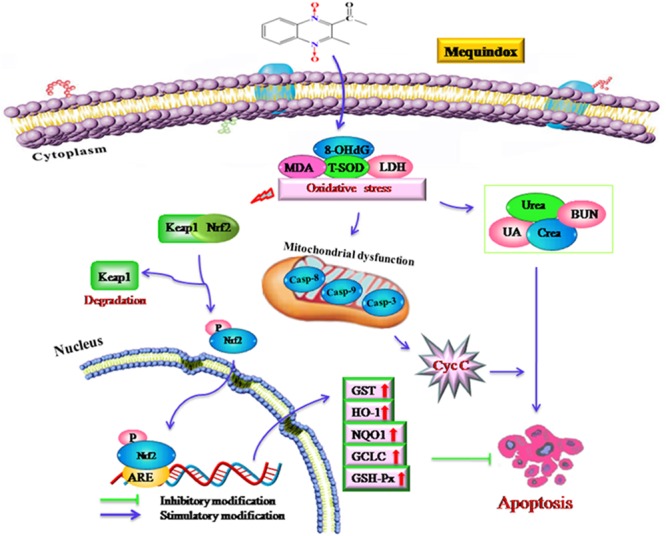
The proposed mechanisms of oxidative stress in mouse after chronic exposure to MEQ. The oxidative stress-imbalance occurs, accompanying the increase of 8-OHdG, MDA, T-SOD, and GSH, which lead to mitochondrial damage and apoptosis in a mitochondrial-dependent apoptotic pathway. The oxidative stress activates Nrf2/Keap1. MEQ upregulated Bax/Bcl-2 ratio, disrupted mitochondrial permeability transition pores, and subsequently, caused cytochrome c release and a cascade activation of caspase, eventually induced apoptosis.

Apoptosis can be triggered by oxidative stress and the damage to DNA (Skipper et al., 2016). MEQ was reported to induce DNA damage in testis of rats *in vivo*, and increased the level of 8-OHdG in rats (Ihsan et al., 2011) and mice (Liu et al., 2017). In this study, the apoptosis in kidney might be resulted from the oxidative stress and mitochondrial dysfunction mediated by MEQ. Currently, there are two well-characterized caspase-activating cascades: one is initiated by the cell surface death receptor and the other is triggered by changes in mitochondrial integrity (Alabsi et al., 2016). Mitochondria were reported to be a target organelle of QdNOs and extra production of ROS might be result in mitochondrial damage, following with mitochondrial apoptotic pathway (Dai et al., 2015). Herein, the organelle included mitochondrial showed vacuolization in the MEQ-treated group, demonstrating the destroyed integrity of mitochondria caused by MEQ. Our data revealed that the mitochondrial damage was induced by MEQ, and the oxidative stress and mitochondrial dysfunction might lead to apoptosis in the kidney of mice after administration of MEQ for a long period (**Figure 8**). Further study should be conducted to investigate the role of mitochondria in the oxidative stress and apoptosis after chronic exposure to MEQ.

The Nrf2-Keap1 pathway was a major cellular defense mechanism against oxidative stress via encoding antioxidant enzymes and phase II detoxifying enzymes (Kobayashi and Yamamoto, 2005; Kimura et al., 2007; Li and Kong, 2009; Pedruzzi et al., 2012). Previous studies suggested that the Nrf-2 was involved in the oxidative damage caused by QCT in rat’s liver (Yang et al., 2013; Yu et al., 2013) and NCI-H295R cells (Wang et al., 2016a). Herein, our results demonstrated that the mRNA expression of Nrf-2, GST-Px, NQO1, GST, HO-1, and GCLC were significantly upregulated in the M110 group. Accordingly, the marked increased protein expression of Nrf-2 was noted in MEQ-treated groups. Therefore, the Nrf2-Keap1 signaling pathway was activated by MEQ in mice. Subsequently, the phase II detoxifying enzymes (HO-1, GCLC, and NQO1) and antioxidative enzymes (GST-Px, GST) were generated and these enzymes eventually protected cells from oxidative stress. Interestingly, the mRNA levels of HO-1 and GCLC were significant decreased accompanying the increased of Nrf-2 at the M25 and M55 groups, indicating that the activation of HO-1 and GCLC by Nrf-2 was inhibited, and the further investigation into this phenomenon was needed. Taken together, current study findings revealed that the Nrf2-Keap1 signaling pathway was activated by MEQ, which may be responsible for the protective effect against oxidative damage, apoptosis, and the necrosis in mouse kidney induced by MEQ (**Figure 8**). Therefore, it was suspected that the Nrf2-Keap1 family was involved in the kidney toxicity caused by MEQ *in vivo*.

Scientists in 2013 reported that the exposure of rats to QCT for 4 weeks with high dose of QCT (2400 mg/kg b.w.) results in significant increased expression of Nrf2 protein in kidney along with the oxidative stress (Yu et al., 2013). However, after rats were treated with QCT for 13 weeks with high dose of QCT (2400 mg/kg b.w.) results in significantly reduced expression of protein Nrf2 and HO-1 along with excessive ROS generation and irreversible oxidative DNA damage, inflammation, and apoptosis in liver, indicated that persistent QCT exposure will inhibit the expression of Nrf2 and HO-1, and aggravate hepatocyte damage (Yu et al., 2014). These findings also suggested a hormesis in the chronic liver disease. Hormesis is a dose-response phenomenon characterized by a low-dose stimulation and a high-dose inhibition (Calabrese et al., 2010). In the present study, a few toxicity-linked parameters, such as serum urea, caspase-8, and GSH, were changed by MEQ not in a dose-dependent manner. It was well documented that the hormetic dose responses were mediated for endogenous cellular defense pathways, including sirtuin and Nrf2 pathways (Calabrese et al., 2010). Therefore, hormesis was suspected to account for these results, and the further study needed to clarify this hypothesis.

## Conclusion

As shown in **Figure 8**, the current study demonstrated that the kidney was an important toxicity organ target of MEQ *in vivo*, and the oxidative stress and mitochondrial damage were induced in mice after administration of MEQ for 11 months. Additionally, MEQ upregulated Bax/Bcl-2 ratio, caused cytochrome c release, and a cascade activation of caspase, eventually induced apoptosis. Our findings revealed that the oxidative stress might be responsible for mitochondrial damage and apoptosis in a mitochondrial-dependent apoptotic pathway. Furthermore, the present study illustrated that the Nrf2-Keap1 family played a protective role in MEQ-induced redox imbalance damage in the kidney of mice. These findings may contribute to a better understanding of the molecular organ toxicity of MEQ and other QdNOs *in vivo*.

## Author Contributions

ZY conceived the idea; XW analyzed and discussed the data; QLi analyzed and discussed the data and wrote the paper; ZL performed and revised the experiments; AL performed the experiments; QW and MD revised the paper. All the authors discussed the results and contributed to the final manuscript.

## Conflict of Interest Statement

The authors declare that the research was conducted in the absence of any commercial or financial relationships that could be construed as a potential conflict of interest. The reviewer SS and handling Editor declared their shared affiliation.

## Supplementary Material

FIGURE S1 Selected microphotographs of kidney in M110 group.

FIGURE S2 Protein expression of cleaved-caspase 3 detected by immunohistochemical assays in M25 group.

FIGURE S3 Protein expression of cleaved-caspase 3 detected by immunohistochemical assays in M55 group.

FIGURE S4 Protein expression of cleaved-caspase 3 detected by immunohistochemical assays in M110 group.

FIGURE S5 Protein expression of Nrf-2 detected by immunohistochemical assays in M25 group.

FIGURE S6 Protein expression of Nrf-2 detected by immunohistochemical assays in M55 group.

FIGURE S7 Protein expression of Nrf-2 detected by immunohistochemical assays in M110 group.

## Abbreviations

8-OHdG: 8-hydroxy deoxyguanosine
BUN: blood urea nitrogen
CBX: carbadox
crea: creatinine
CYA: cyadox
GCLC: glutamate-cysteine ligase catalytic subunit
GSH: glutathione
GSH-Px: glutathione peroxidase
GST: glutathione S-transferase
HO-1: hydroxyl radicals
IκB: nuclear factor κB protein
LDH: lactate dehydrogenase
M4: 3-methyl-2-(1-hydroxyethyl) quinoxaline-*N*4-monoxide
M8: 3-methyl-2-(1-hydroxyethyl) quinoxaline-*N*1-monoxide
MDA: malondialdehyde
MEQ: mequindox
*N*1-MEQ: *N*1-desoxymequindox
NQO1: NAD(P)H:quinoneoxidoreductase
OLA: olaquindox
QCT: quinocetone
QdNOs: quinoxaline-di-*N*-oxides
ROS: reactive oxygen species
SOD: superoxide dismutase
TEM: transmission electron microscope
T-SOD: total superoxide dismutase
UA: uric acid
