# Supplementary figures and images for: Mequindox-Induced Kidney Toxicity Is Associated With Oxidative Stress and Apoptosis in the Mouse
Source: Front Pharmacol. 2018 May 1;9:436. doi: 10.3389/fphar.2018.00436 (PMC5938394; doi:10.3389/fphar.2018.00436)

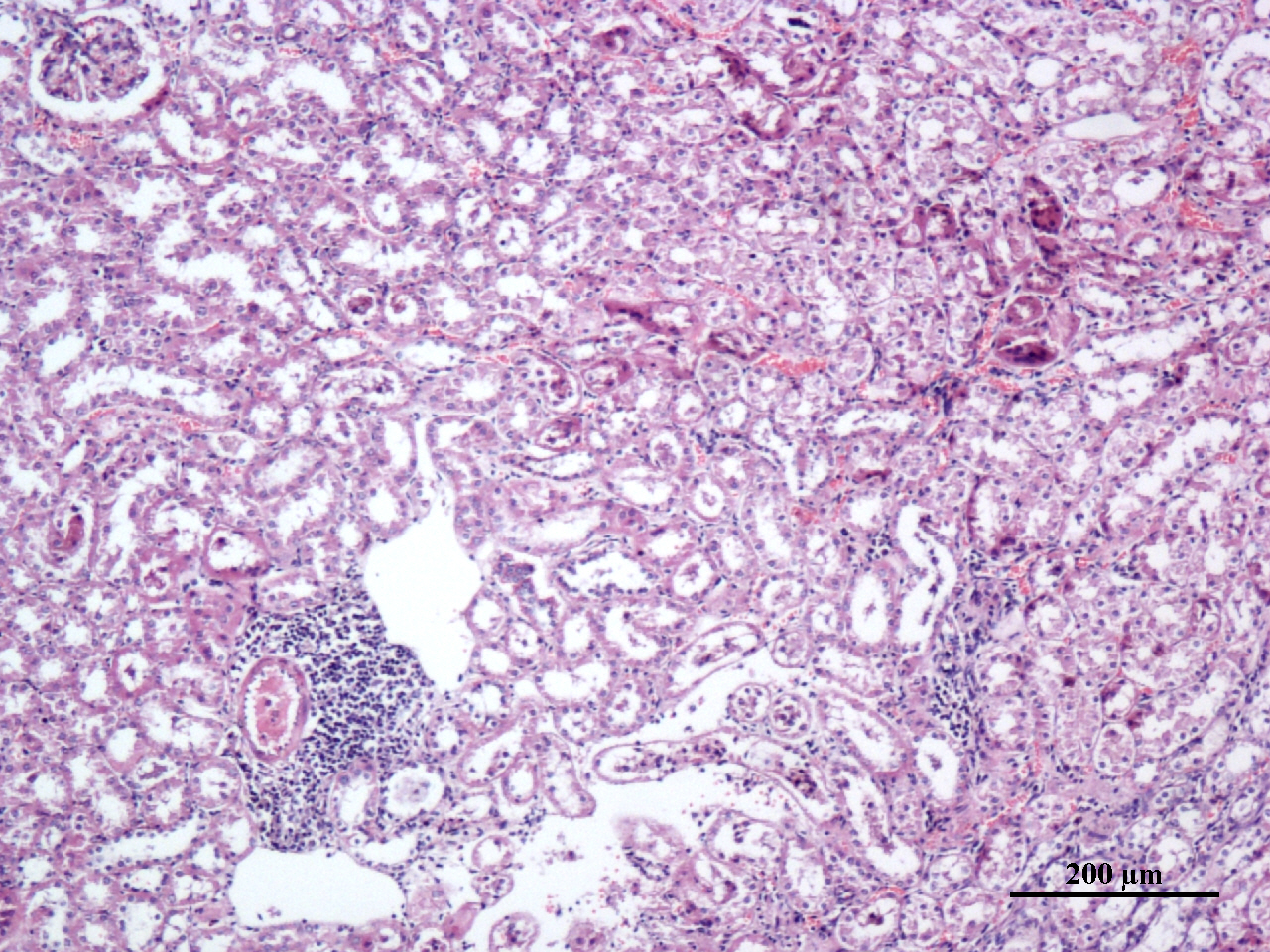

Supplement: FIGURE S1 — Selected microphotographs of kidney in M110 group. [file Image_1.JPEG]

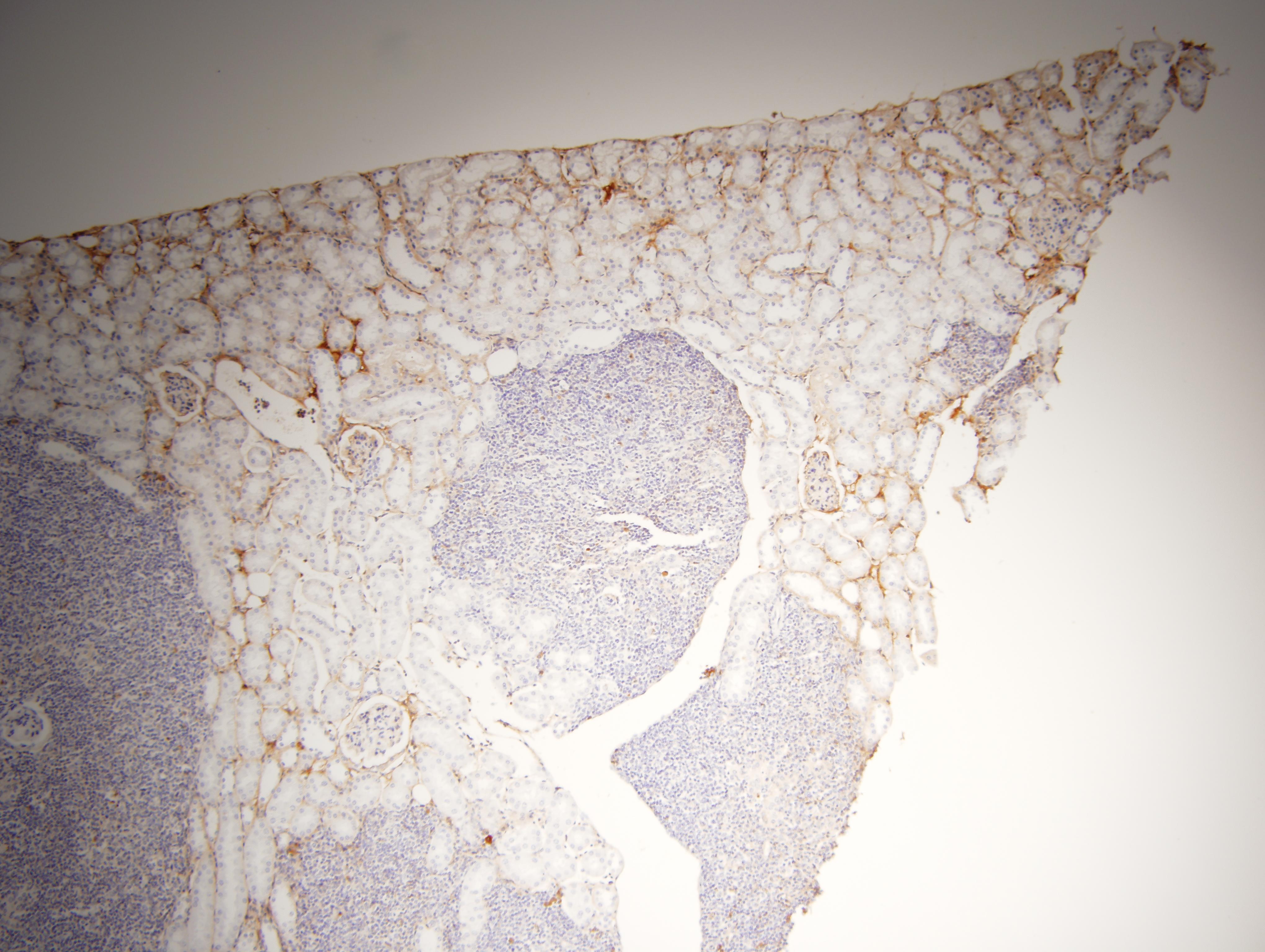

Supplement: FIGURE S2 — Protein expression of cleaved-caspase 3 detected by immunohistochemical assays in M25 group. [file Image_2.JPEG]

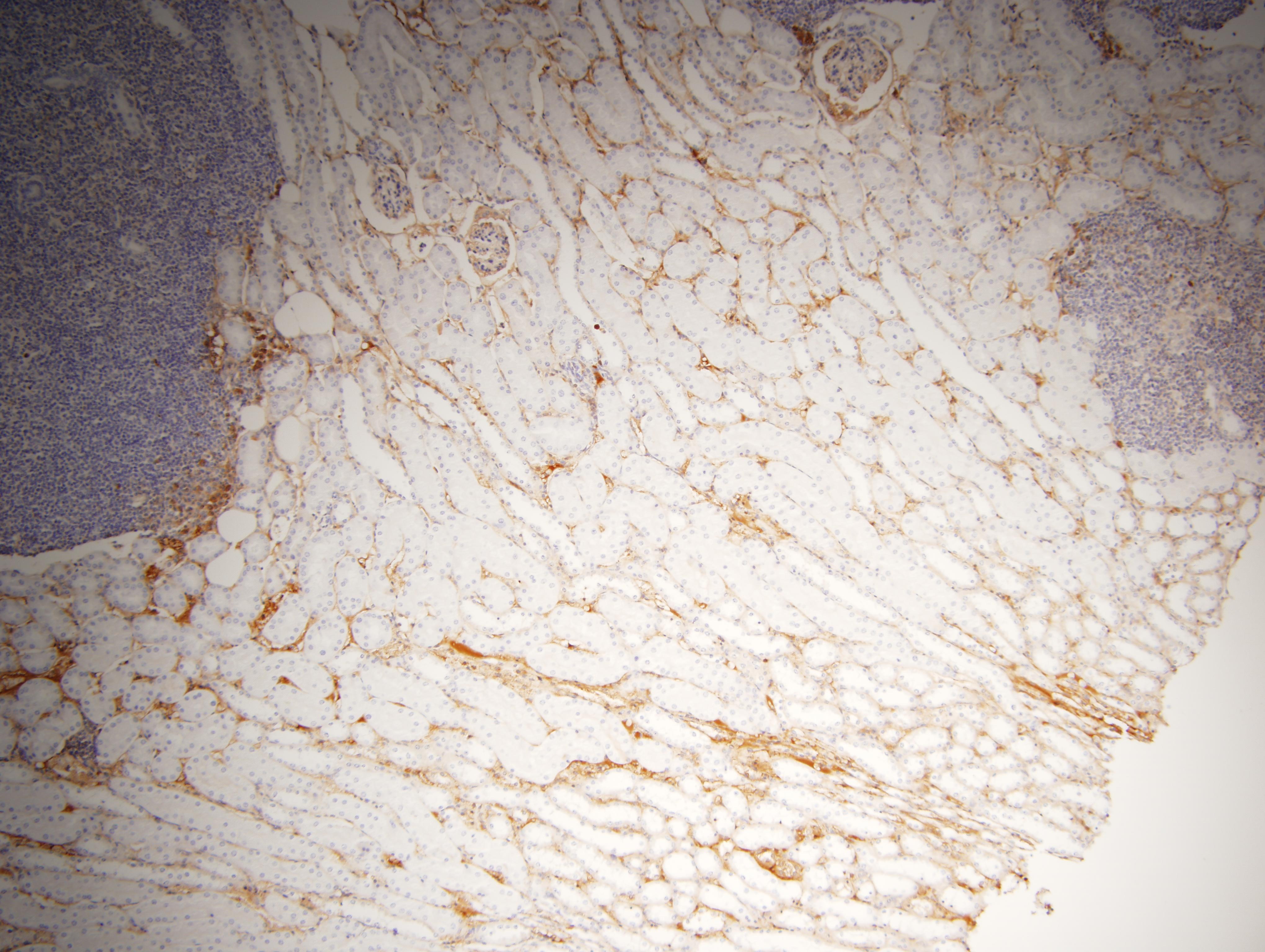

Supplement: FIGURE S3 — Protein expression of cleaved-caspase 3 detected by immunohistochemical assays in M55 group. [file Image_3.JPEG]

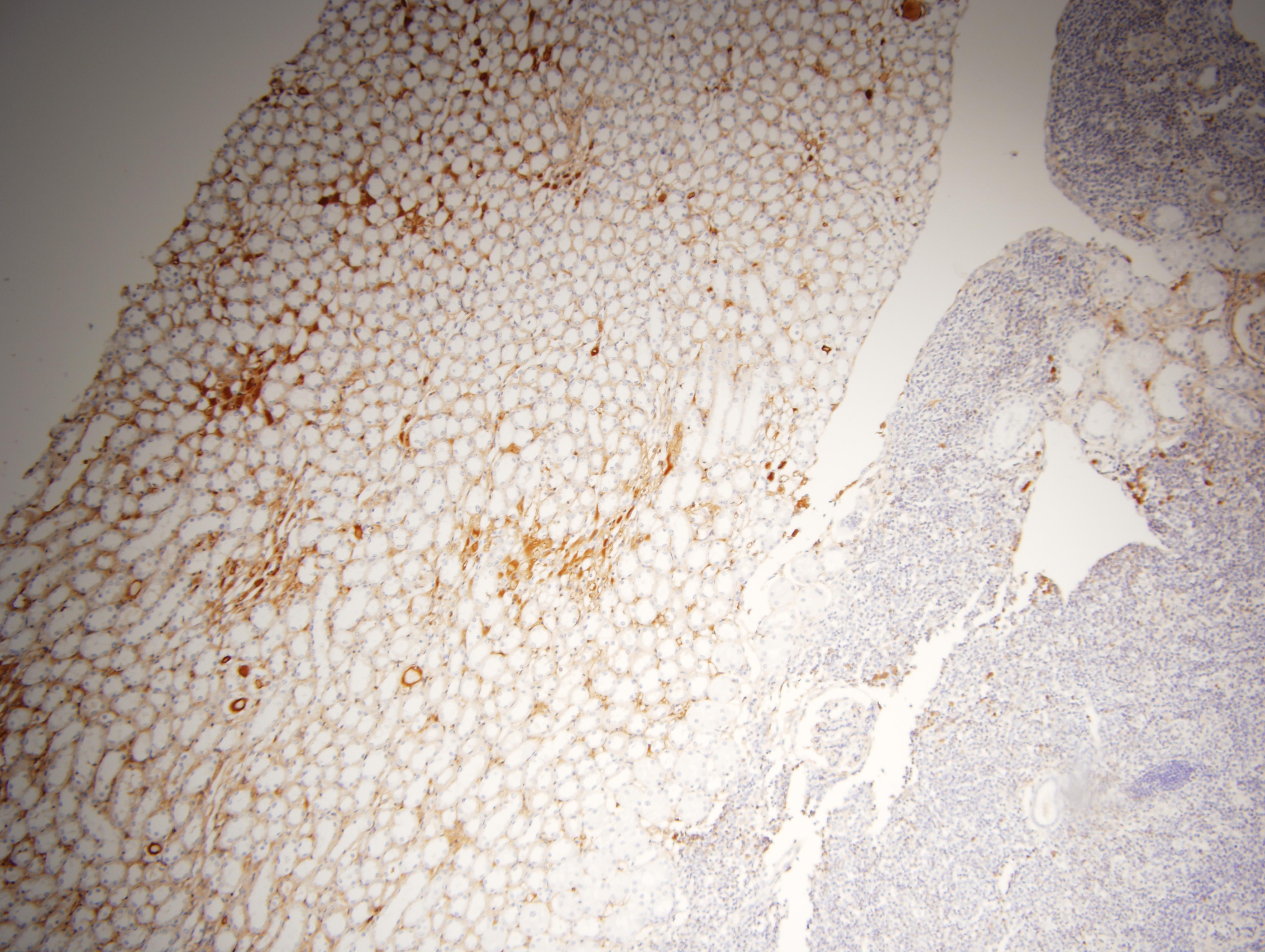

Supplement: FIGURE S4 — Protein expression of cleaved-caspase 3 detected by immunohistochemical assays in M110 group. [file Image_4.JPEG]

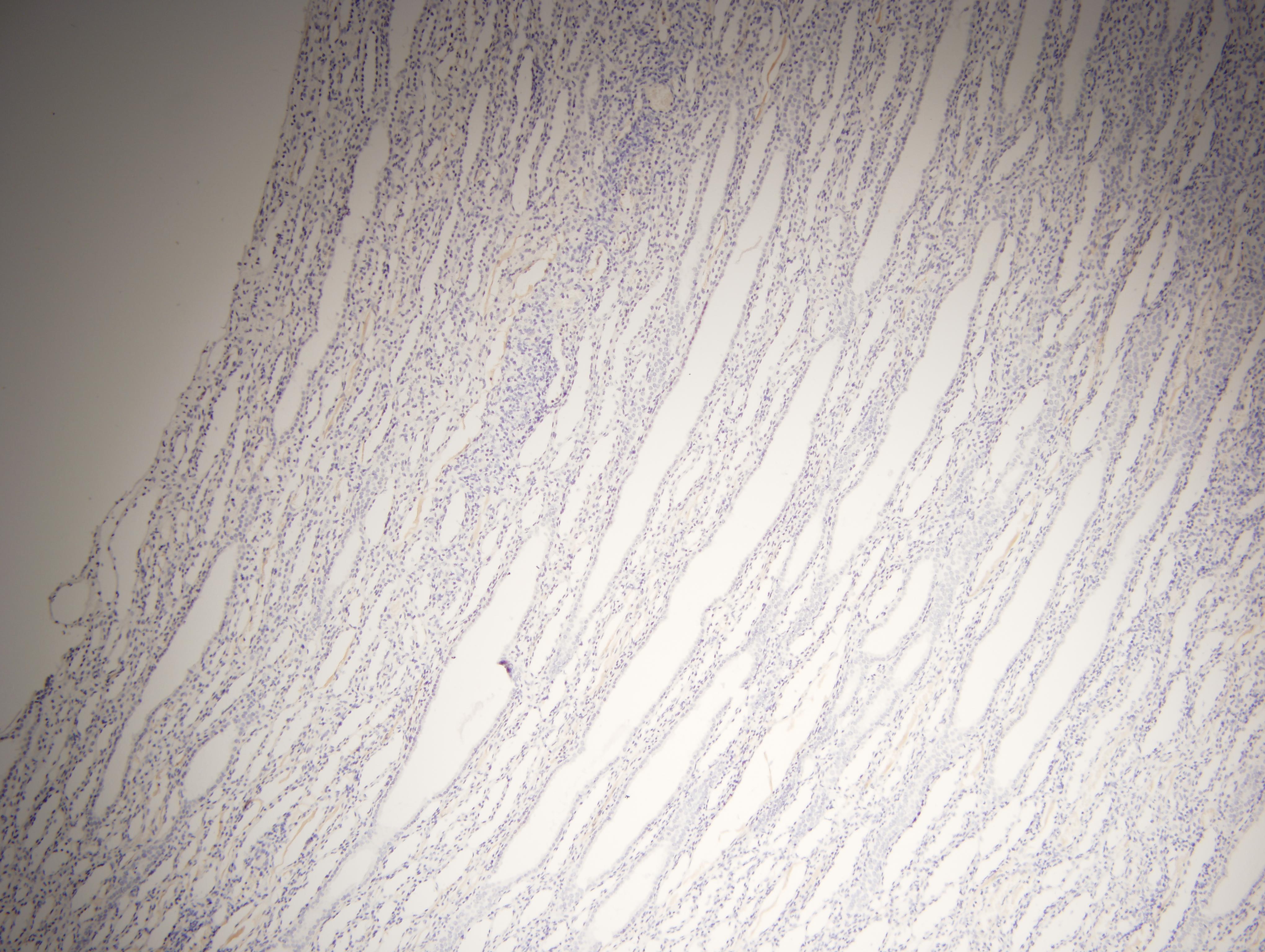

Supplement: FIGURE S5 — Protein expression of Nrf-2 detected by immunohistochemical assays in M25 group. [file Image_5.JPEG]

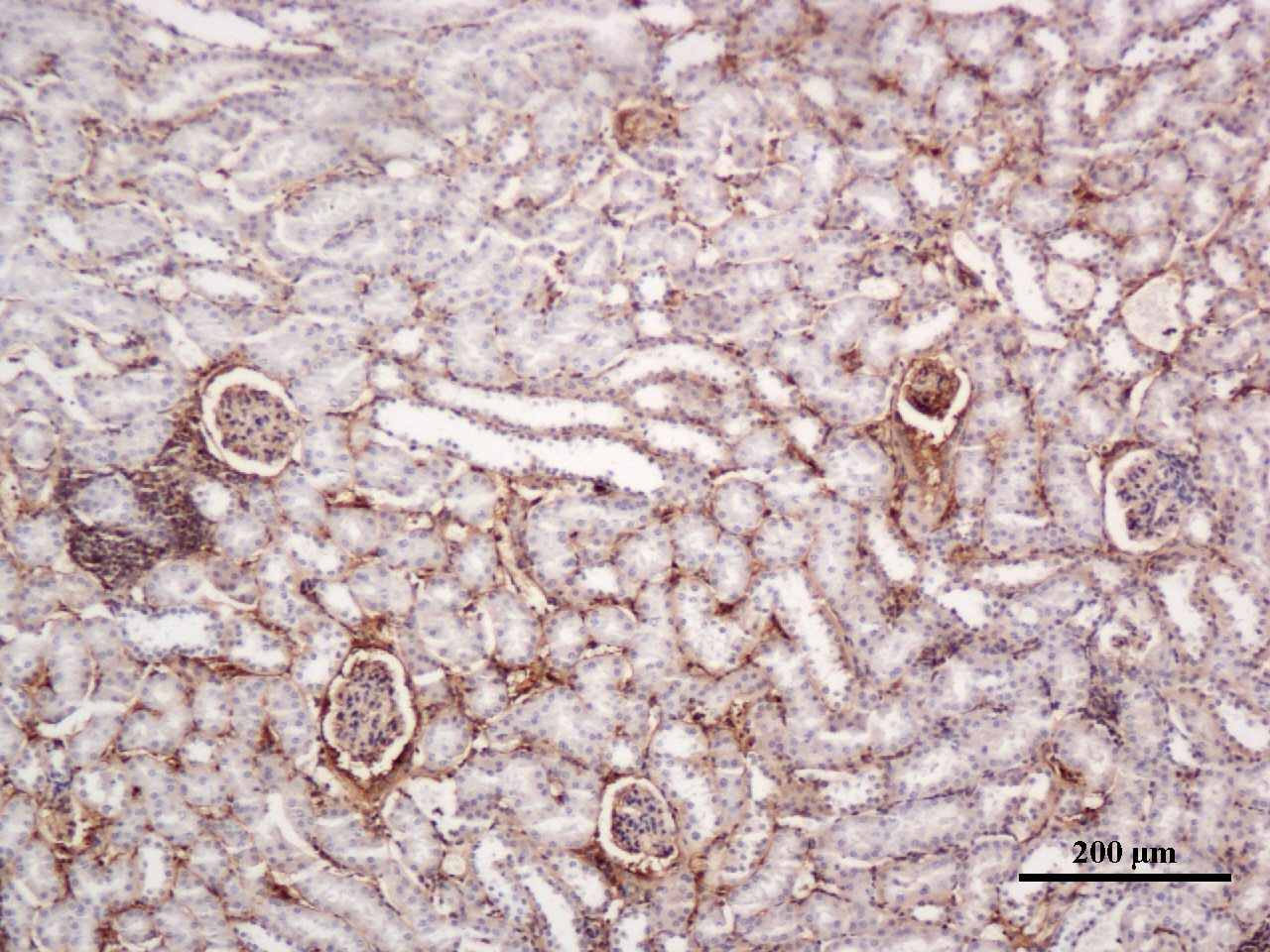

Supplement: FIGURE S6 — Protein expression of Nrf-2 detected by immunohistochemical assays in M55 group. [file Image_6.JPEG]

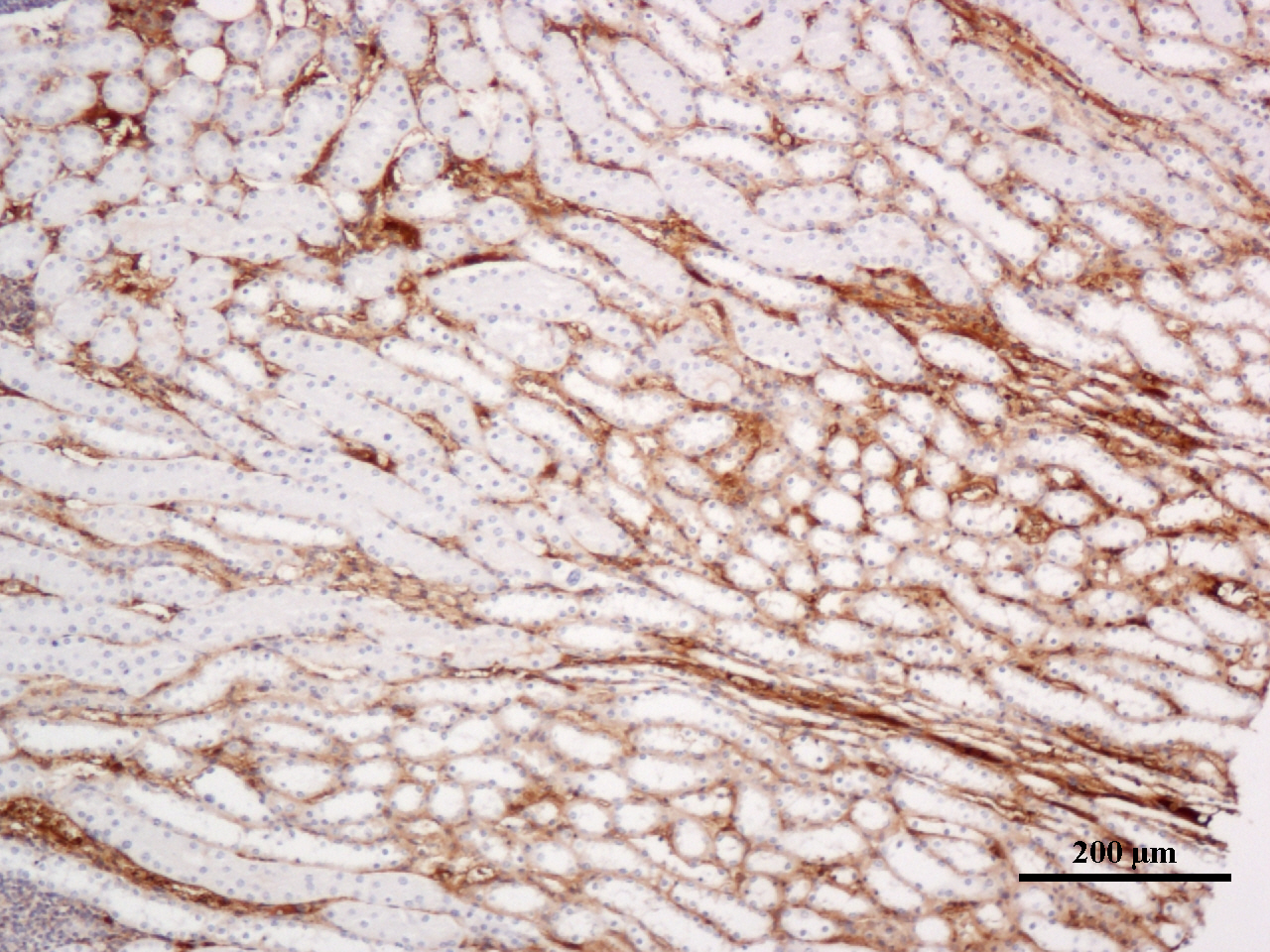

Supplement: FIGURE S7 — Protein expression of Nrf-2 detected by immunohistochemical assays in M110 group. [file Image_7.JPEG]
